# Advancements and Complexities in the Conversion of Lignocellulose Into Chemicals and Materials

**DOI:** 10.3389/fchem.2020.00797

**Published:** 2020-08-21

**Authors:** Giulia Fiorani, Claudia Crestini, Maurizio Selva, Alvise Perosa

**Affiliations:** ^1^Department of Molecular Sciences and Nanosystems, Ca' Foscari University of Venice, Venice, Italy; ^2^C4S Center for Sustainability, Ca' Foscari University Foundation, Calle Larga Ca' Foscari, Venice, Italy

**Keywords:** plant-based biomass, biobased platform chemicals, levulinic acid, dimethyl carbonate, lignin valorization, lignin characterization, lignin fractionation, lignin-based nanomaterials

## Abstract

This Perspective describes the challenges and objectives associated to the development of new chemical technologies for the conversion of lignocellulose (non-food or waste) into chemicals and materials; it also provides an outlook on the sources, potential products, and issues to be addressed.

## Introduction

Plant-based biomass plays a pivotal role in the development of economically and environmentally sustainable biorefinery processes. Three different biopolymers are included in lignocellulosic biomass, namely cellulose, hemicellulose, and lignin, which are characterized by different chemical composition and reactivity. The chemical diversity of raw biomass represents a challenge toward the development of energy and resource efficient chemical processes and of the associated technological tools (Xu et al., 2019). For example, most of 50–70 MT/year of lignin produced by both the pulp and paper industry and modern saccharification processes are currently employed in low added-value applications (e.g., burned for energy co-generation) (Luo and Abu-Omar, 2017). Back in 2004, a rational selection of biobased *platform chemicals* was reported and became a strategic tool to develop focused valorization strategies (Werpy and Petersen, 2004); since then, the list of renewable-based platform chemicals and the associated chemical- and biochemical-based valorization strategies is constantly monitored and updated (Bozell et al., 2007; Bozell and Petersen, 2010; Esposito and Antonietti, 2015; Lee et al., 2019; Huo and Shanks, 2020). Currently, a plethora of commercial cellulose and hemicellulose valorization processes are available (Aresta et al., 2015), while examples of integrated biorefinery processes were reported only recently (BBI JU Annual Activity Report, 2019; Liao et al., 2020).

This Perspective showcases some recent examples of (i) preparation of selected building blocks derived from established biobased platform chemicals [e.g., levulinic acid (LVA) and OH-bearing biobased derivatives (BBDs) ] and (ii) non-destructive technologies for the valorization of lignin. For both classes of biobased chemicals, valorization occurred employing mild, eco-friendly technologies.

## LVA Hydrogenation

**Figure 1:**
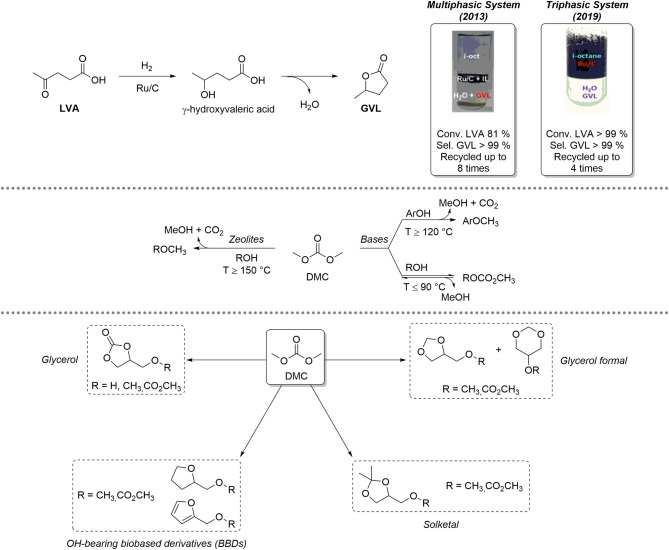
**Top:** multiphasic systems based on Ru/C active for the reduction of LVA to GVL; **center:** dimethyl carbonate (DMC) reactivity; **bottom:** reactivity of DMC and OH-BBDs.

LVA is an important renewable-based platform chemical, which can be obtained selectively upon acidic hydrolysis of polysaccharides (Bozell and Petersen, 2010; Kang et al., 2018). LVA is characterized by a significant synthetic potential in different fields of applications: for example, it is employed as intermediate for the preparation of drugs bearing heterocyclic scaffolds but can also be used as co-monomer for the preparation of renewable-based materials (Esposito and Antonietti, 2015; Adeleye et al., 2019). LVA can be selectively reduced to γ-valerolactone (GVL), which is a low-toxicity, biodegradable five-membered ring heterocyclic compound employed as a fully renewable-based aprotic solvent, fuel additive, and precursor for added-value chemicals (Alonso et al., 2013; Mellmer et al., 2014). LVA reduction to GVL is a sequential process composed of two steps by which LVA is initially hydrogenated to the intermediate γ-hydroxyvaleric acid that, in turn, undergoes a dehydration/cyclization reaction to give GVL (Figure 1, top). These transformations typically occur in solution in presence of homogeneous metal complexes based on Ru, Ir, Pd, and, more recently, Fe (Omoruyi et al., 2016). Nevertheless, GVL recovery by distillation is non-practical and anti-economical, due to its high boiling point (T_eb_ = 207–208°C). Consequently, heterogenized Ru-based catalysts were developed, including complexes with sulfonated ligands for effective confinement in the aqueous phase, and/or Ru-based catalysts supported on mesoporous or amorphous materials (Wright and Palkovits, 2012). Performing LVA hydrogenation in multiphasic systems (MPs) represents a promising strategy to improve selective GVL formation as well as catalyst recovery. A MP consisting of three immiscible phases (e.g., water, an apolar solvent, *iso*-octane, and an ionic liquid, IL) was initially proposed. The catalyst (Ru/C) was effectively segregated in the IL phase and recycled up to eight times without losing its performance; in all catalytic runs, GVL was formed quantitatively (LVA Conv. = 81%; GVL Sel. > 99%) and exclusively in the aqueous solution (Selva et al., 2013). More recently, Ru/C catalyzed quantitative conversion of LVA to GVL was observed even in a simple biphasic H_2_O/*iso*-octane system. In the absence of any IL, the catalyst could be selectively confined (suspended) in the hydrocarbon medium, on condition that the aqueous solution was acidic in a pH range of 2.5–3. Ru-leaching in water was neglectable (Ru < 0.01% w/w) (Bellè et al., 2019).

## Valorization of Oh-Bearing BBDs With Dimethyl Carbonate (DMC)

The lightest term of the dialkyl carbonates series, dimethyl carbonate (DMC), has an established role as a low environmental impact reagent and solvent (Fiorani et al., 2018; Selva et al., 2019). DMC embeds different non-equivalent electrophilic groups within its structure (one *sp*^2^ carbonate C and two *sp*^3^ hybridized methyl C) and can therefore react as an ambident reagent for selective carboxymethylation and/or methylation of a variety of *O*-, *S*- *C*-, *N*-, and *P*-based nucleophiles (e.g., alcohols, phenols, methylene active compounds, amines, and phosphines). Figure 1, center, exemplifies the case of alcohols and phenols. At low temperatures (*T* ≤ 90°C) and in the presence of base catalysts, only transcarbonation reactions take place via a B_Ac_2 mechanism: equilibrium product (ROCOOMe) formation is favored by continuous removal of MeOH via azeotropic distillation with DMC or by adding suitable adsorbing porous materials (molecular sieves, zeolites, etc.). At higher temperatures (*T* > 120–150°C) and in the presence of weak bases or amphoteric catalysts like alkali metal exchanged faujasites, methylation occurs selectively following a B_Al_2 mechanism. In the latter case, methylation products (ArOCH_3_) are formed irreversibly with release of CO_2_. Within our long-lasting interest in eco-compatible processes using renewable-based starting materials, our group has developed a solid expertise on the use of DMC for the selective chemical upgrading of biosourced platform chemicals, as depicted in Figure 1, bottom. In-depth chemical valorization studies have been carried out by us on various OH-BBDs, including glycerol (Glyc), its cyclic acetals solketal and glycerol formal and other bioderived aliphatic alcohols. DMC-based protocols allowed for the selective preparation of OH-BBDs methyl ether derivatives, which find applications as fuel additives as well as solvents and chemical intermediates (Rorrer et al., 2019) or for the synthesis of symmetrical aliphatic dialkyl carbonates, which are rapidly gaining importance and expanding the range of applications as biobased polar aprotic solvents (Mao et al., 2019). For instance, the reactivity of Glyc and DMC under thermal (catalyst-free) conditions was thoroughly studied: (i) in batch mode, glycerol carbonate methyl ether was obtained selectively when working in large DMC excess (DMC/Glyc = 60:1 mol/mol, *T* = 180°C, *t* = 5 h, yield = 82%). Interestingly, under a CO_2_ atmosphere [DMC/Glyc = 20:1 mol/mol, *T* = 180°C, *t* = 5 h, *p* (CO_2_) = 20 bar], the reaction led to the formation of glycerol carbonate in up to 84% yield; (ii) in continuous-flow (CF) mode (DMC/MeOH/Glyc = 10:6:1 mol/mol, *p* = 50 bar, *F* = 0.1 ml·min^−1^, *T* = 230–250°C); instead, glycerol carbonate was achieved in up to 92% yield at *T* = 230°C (Guidi et al., 2016). The CF-reaction of DMC with OH-BBDs was further explored using weakly basic hydrotalcite catalysts (HTs). *O*-alkylation, with formation of the corresponding methyl ethers (> 99% yield) was the preferred pathway (DMC/ROH = 20:1 to 5:1 mol/mol, *p* = 1 bar, *F* = 0.1 ml·min^−1^, *T* = 150–260°C) (Cattelan et al., 2017). Interestingly, HT catalysts displayed a high activity and selectivity also for the preparation of symmetrical dialkyl carbonates via a two-step carbonate interchange reaction (CIR). In this case, alkyl methyl carbonate intermediates were initially formed by batch reaction of various alcohol(s) with DMC at *T* = 90°C. Thereafter, intermediates were converted into the desired symmetrical carbonates through disproportionation reactions carried out under CF conditions at high *T* (*T* = 180–275°C) (Cattelan et al., 2018).

## Lignin

**Figure 2:**
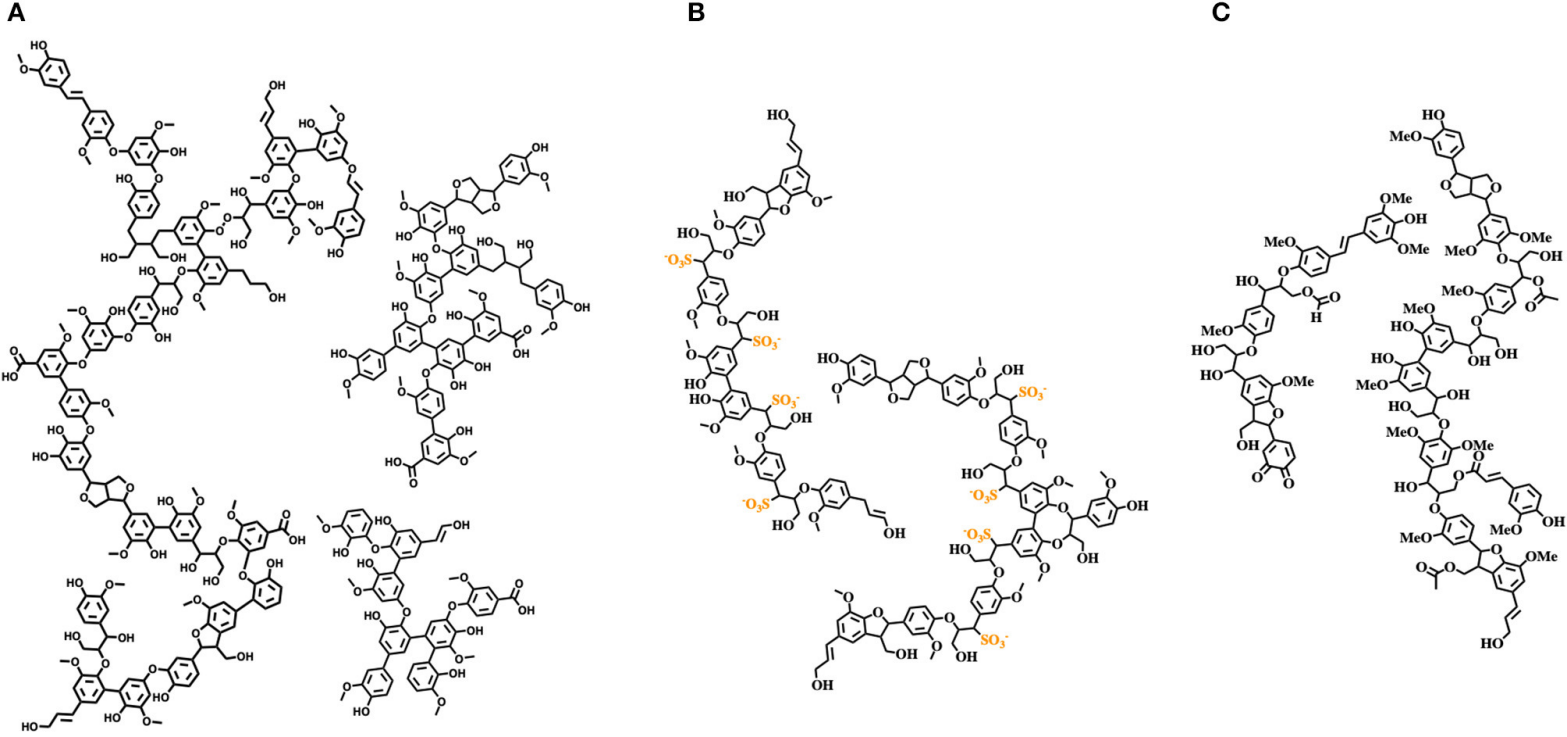
Structures of relevant technical lignins. **(A)** Softwood kraft lignin; **(B)** softwood lignosulfonate; **(C)** wheat straw organosolv lignin.

The potential of lignin as a feedstock is enormous due to its abundance and rich chemical nature, consisting mainly of aromatic and phenolic subunits. Different valorization pathways have been explored, such as direct lignin valorization, i.e., development of innovative renewable-based materials, or chemical transformation in aromatic commodities and chemicals such as for example, in the “lignin first” approach consisting in reductive treatments of biomass yield complex mixtures of partially reduced lignin derivatives useful for biofuel production (Sun et al., 2018). Nevertheless, industrially and commercially relevant lignin valorization processes are still needed (Argyropoulos and Crestini, 2016; Sun et al., 2018). Lignin stream valorization is hampered by two main factors: (i) lignin is an extremely complex biomaterial lacking a defined primary structure, with a specific composition severely affected by the botanical origin and location, altering monomers' ratio and their linking modes; (ii) industrially available lignins are highly variable heterogeneous, polyfunctional complex mixtures of unpredictable specifications, with distinct physicochemical properties compared to native lignin, largely due to the different processes required for their isolation (Figure 2). Therefore, to fully exploit lignin streams as feedstocks for further utilization, they should initially be refined to reproducible “cuts” with consistent specifications. At the same time, structural characterization studies and development of *ad hoc* analytical techniques are vibrant and challenging research topics useful to accelerate the development of circular lignin valorization value chains (Sette et al., 2011; Meng et al., 2019). For example, the structural features of milled wood lignin (MWL, which strongly resembles native lignin) were elucidated only in 2011 by an array of NMR techniques, unambiguously showing that MWL is a linear oligomer rather than a highly branched polymer (Crestini et al., 2011). Structural elucidation studies performed on softwood kraft lignin highlight the presence of two different components: one derived from native lignin and the other composed of repolymerized oligomeric fragments generated during the Kraft pulping process (Crestini et al., 2017). Given the high variability and diversity of commercially available lignin streams, design and development of fractionation protocols for the isolation of distinct lignin fractions characterized by the same molecular weight distribution and chemical properties represent a key purification technology. Several studies on lignin fractionation have been reported, mainly relying on fractional precipitation and/or sequential dissolution in the presence of solvents with different polarity, aqueous solutions at different pHs, or membrane filtration (Cui et al., 2014; Sevastyanova et al., 2014; Duval et al., 2016; Lange et al., 2016). Lignin fractionation opens the door to a more widespread exploitation of commercial lignin stream derivatives in materials science. Moreover, specific fractions can be selectively modified (e.g., varying solubility, hydrophobicity, surface adhesion, antioxidant activity, UV screening, antimicrobial activity, anti-inflammatory activity, etc.). Development of accessible and reproducible tailoring processes will promote lignin inclusion in a large variety of consumer products, i.e., home and/or personal care products (Brooker et al., 2016a,b), composites, packaging materials coatings, and resins, retaining the desired macroscopic properties and, at the same time, mitigating the overall environmental footprint and improving their biodegradation.

The development of innovative materials derived from biomass is a timely fundamental research challenge. To this aim, in recent years, several different renewable-based micro- and nanostructured materials were developed and successfully applied, among others, in microelectronics, cosmetics, nutraceutical, and pharmaceutical applications. Lignin nanoparticles were initially developed for agricultural applications as vectors for the controlled release of active principles, or in nanocomposites formulation (Tortora et al., 2014; Bartzoka et al., 2016; Sipponen et al., 2018). However, thanks to their high biocompatibility, lignin microcapsules can also be employed for the controlled and synergic release of pharmaceutical and/or cosmetic active principles and for the design and development of functional foods. The range of potential applications of lignin-based nanomaterials is expanding continuously and now also includes preparation of renewable-based lignin nanofibers suitable for carbon nanofiber production and use in structural composites and energy storage applications (Kumar et al., 2019).

## Conclusions

Plant-based biomass plays a pivotal role in the development of economically and environmentally sustainable biorefinery processes. The chemical complexity of plant biomass, however, still represents a challenge toward the development of energy- and resource-efficient chemical processes and of the associated technological tools. Reliable convergent chemical strategies enabling transformations of biomass-derived matrices in discrete families of platform chemicals will be crucial to improve the biorefinery efficiency. This Perspective article, showcasing some recent examples of valorization of biopolymers and platform chemicals derived from lignocellulosic biomass, aims at offering the Reader the scenario of issues associated to the implementation of new chemical technologies for the conversion of lignocellulose (non-food or waste) into chemicals and materials; at the same time, it also provides an outlook on the sources and potential products to be addressed using multiphase systems, eco-compatible reagents like DMC, and the design of protocols for lignin fractionation. Notably, this approach should be complemented with the advance of analytical techniques for the identification of the most promising added-value structures of a given valorization process.

## Author Contributions

All authors contributed to writing and revising the manuscript. All authors have read and agreed the final version of the Perspective.

## Conflict of Interest

The authors declare that the research was conducted in the absence of any commercial or financial relationships that could be construed as a potential conflict of interest.
